# Supplementary material for: Toward computing attributions for dimensionality reduction techniques
Source: Bioinform Adv. 2023 Aug 3;3(1):vbad097. doi: 10.1093/bioadv/vbad097 (PMC10502234; doi:10.1093/bioadv/vbad097)
Supplement: vbad097_Supplementary_Materials [file vbad097_supplementary_materials.pdf]

A. Derivation of t-SNE Gradients

We derive the equations for the gradient of the t-SNE (van der Maaten and Hinton, 2008) output embedding with respect to the input data. Suppose we have datapoints $x_1, ..., x_N \in \mathbb{R}^D$. We denote $x_{i,d}$ as the dth feature of the ith datapoint. Within the context of supervised classification, the gradient attribution method (Simonyan et al., 2014) is defined as follows. For a given score function of class c: $S_c(x) \in \mathbb{R}$, we define the attribution for x_i as:

$$A_c(x_i) = \left[\frac{\partial S_c(x)}{\partial x_{i,1}}, \cdots, \frac{\partial S_c(x)}{\partial x_{i,D}}\right] \tag{10}$$

The t-SNE algorithm is a dimensionality reduction technique. Given our data, it will return embeddings of dimension $C < D$. We can think of our output dimension as a score function, so we can write:

$$t - SNE(x_1, ...x_N) = \left\{\begin{bmatrix} S_1(x_1) \\ \vdots \\ S_C(x_1) \end{bmatrix}, \cdots, \begin{bmatrix} S_1(x_N) \\ \vdots \\ S_C(x_N) \end{bmatrix}\right\} \tag{11}$$

To keep the notation consistent with the original t-SNE paper, we denote $y_{i,c} = S_c(x_i)$ and $y_i = [y_{i,1}, \cdots y_{i,C}]$. The t-SNE function is iterative (over steps $1, \cdots, T$). We denote the output embedding at step t as y_i^t. Ignoring the optimisation terms, we have that:

$$y_{i,c}^t = y_{i,c}^{t-1} + dy_{i,c}^t \tag{12}$$

Where:

$$dy_{i,c}^t = 4\sum_{j \neq i}(p_{i,j} - q_{i,j}^{t-1})\phi_{i,j,c}^{t-1} \tag{13}$$

$$\phi_{i,j,c}^{t-1} = (y_{i,c}^{t-1} - y_{j,c}^{t-1})(1 + ||y_i^{t-1} - y_j^{t-1}||^2)^{-1} \tag{14}$$

In this set-up, we notice that we could compute:

$$\frac{\partial y_{i,c}^t}{\partial x_{j,d}} \quad i, j \in \{1, \cdots, N\}, \quad c \in \{1, \cdots, C\}, \quad d \in \{1, \cdots, D\}, \quad t \in \{1, \cdots, T\} \tag{15}$$

We restrict our interest to **only gradients of an embedding with respect to their corresponding input data point**:

$$\frac{\partial y_{i,c}^t}{\partial x_{i,d}} = \frac{\partial y_{i,c}^{t-1}}{\partial x_{i,d}} + \frac{\partial dy_{i,c}^t}{\partial x_{i,d}} \tag{16}$$

We use the chain rule on 13 to obtain:

$$\frac{\partial dy_{i,c}^t}{\partial x_{i,d}} = 4\sum_{j \neq i}\left\{\left(\frac{\partial p_{i,j}}{\partial x_{i,d}} - \frac{\partial q_{i,j}^{t-1}}{\partial x_{i,d}}\right)\phi_{i,j,c}^{t-1} + (p_{i,j} - q_{i,j})\frac{\partial \phi_{i,j,c}^{t-1}}{\partial x_{i,d}}\right\} \tag{17}$$

At step $t-1$, we store $\frac{\partial y_{i,c}^{t-1}}{\partial x_{i,d}}$ so it can be accessed at step t. This allows us to compute the following:

$$\frac{\partial q_{i,j}^{t-1}}{\partial x_{i,d}} = \sum_{c'=1}^{C} \frac{\partial q_{i,j}^{t-1}}{\partial y_{i,c'}^{t-1}} \frac{\partial y_{i,c'}^{t-1}}{\partial x_{i,d}} \tag{18}$$

$$\frac{\partial \phi_{i,j,c}^{t-1}}{\partial x_{i,d}} = \sum_{c'=1}^{C} \frac{\partial \phi_{i,j,c}^{t-1}}{\partial y_{i,c'}^{t-1}} \frac{\partial y_{i,c'}^{t-1}}{\partial x_{i,d}} \tag{19}$$

We now derive the gradients for $\frac{\partial p_{i,j}}{\partial x_{i,d}}$, $\frac{\partial q_{i,j}^{t-1}}{\partial y_{i,c}^{t-1}}$ and $\frac{\partial \phi_{i,j,c}^{t-1}}{\partial y_{i,c}^{t-1}}$. From the t-SNE paper:

$$p_{i,j} = \frac{p_{i|j} + p_{j|i}}{2N} \quad i \neq j \text{ and } p_{i,i} = 0 \tag{20}$$

So we must differentiate w.r.t. both components. If we let $d_{ij} = \frac{-(x_i - x_j)}{\sigma_i^2}$ [3] and $g_j(x_i) = \exp\left(\frac{-||x_i - x_j||^2}{2\sigma_i^2}\right)$, then:

$$p_{j|i} = \frac{g_j(x_i)}{\sum_{k\neq i} g_k(x_i)} \quad p_{i|j} = \frac{g_i(x_j)}{\sum_{k\neq j} g_k(x_j)} \tag{21}$$

We can apply the quotient rule to differentiate this:

$$\frac{\partial p_{j|i}}{\partial x_i} = \sum_{k \neq i} \frac{d_{ij} g_j(x_i) g_k(x_i)}{\left(\sum_{l\neq i} g_l(x_i)\right)^2} - \sum_{k \neq i} \frac{d_{ik} g_j(x_i) g_k(x_i)}{\left(\sum_{l\neq i} g_l(x_i)\right)^2} \tag{22}$$

$$= d_{ij} p_{j|i} \sum_{k\neq i} p_{k|i} - \sum_{k\neq i} d_{ik} p_{k|i} p_{j|i} \tag{23}$$

$$= p_{j|i}(d_{ij} - \mathbb{E}_{p_{k|i}}[d_{ik}]) \tag{24}$$

$$\frac{\partial p_{i|j}}{\partial x_i} = \sum_{k \neq j} \frac{d_{ji} g_i(x_j) g_k(x_j)}{\left(\sum_{k\neq j} g_k(x_j)\right)^2} - \frac{d_{ji} g_i(x_j)^2}{\left(\sum_{k\neq j} g_k(x_j)\right)^2} \tag{25}$$

$$= \sum_{k\neq j} d_{ji} p_{i|j} p_{k|j} - d_{ji} p_{i|j}^2 \tag{26}$$

$$= d_{ji} p_{i|j}(1 - p_{i|j}) \tag{27}$$

We next derive $\frac{\partial q_{i,j}^{t-1}}{\partial y_{i,c}^{t-1}}$

$$q_{i,j}^{t-1} = \frac{(1 + ||y_i^{t-1} - y_j^{t-1}||^2)^{-1}}{\sum_{k\neq l}(1 + ||y_k^{t-1} - y_l^{t-1}||^2)^{-1}} \quad i \neq j \text{ and } q_{i,i}^{t-1} = 0 \tag{28}$$

Now let $g_j(y_i) = (1 + ||y_i - y_j||^2)^{-1}$ then, using the quotient rule (suppressing unneeded indices):

$$\frac{\partial q_{i,j}}{\partial y_{i,c}} = \frac{-2(y_{i,c} - y_{j,c}) g_j(y_i)^2 \sum_{k\neq l} g_l(y_k)}{(\sum_{k\neq l} g_l(y_k))^2} - \frac{g_j(y_i)(-4)\sum_{k\neq i}(y_{i,c} - y_{k,c}) g_k(y_i)^2}{(\sum_{k\neq l} g_l(y_k))^2} \tag{29}$$

$$= -2q_{ij} g_j(y_i)(y_{i,c} - y_{j,c}) - 2q_{ij}\sum_{k\neq i} -2q_{ik} g_k(y_i)(y_{i,c} - y_{k,c}) \tag{30}$$

$$= -2q_{ij}\left(\phi_{i,j,c} - 2\sum_{k\neq i} q_{ik}\phi_{i,k,c}\right) \tag{31}$$

Finally, for $\phi_{i,j,c}$

[3] Note: σ_i is also a function of the input data. We ignore this relation when computing these gradients.

$$\frac{\partial \phi_{i,j,c}}{\partial y_{i,c'}} = \begin{cases} \frac{(1+||y_i-y_j||^2)-2(y_{i,c}-y_{j,c})^2}{(1+||y_i-y_j||^2)^2} & c=c' \\ \frac{-2(y_{i,c}-y_{j,c})(y_{i,c'}-y_{j,c'})}{(1+||y_i-y_j||^2)^2} & c\neq c' \end{cases} \quad (32)$$

$$= g_j(y_i)1_{c=c'} - 2\phi_{i,j,c}\cdot\phi_{i,j,c'} \quad (33)$$

B. Derivation of Barnes-Hut Approximated t-SNE Gradient

We show how we can use the Barnes-Hut approximation on the t-SNE gradient (attribution) function. Recall that our attributions can be computed as:

$$\frac{\partial dy_{i,c}^t}{\partial x_{i,d}} = 4\sum_{j\neq i}\Bigg\{\left(\frac{\partial p_{i,j}}{\partial x_{i,d}} - \frac{\partial q_{i,j}^{t-1}}{\partial x_{i,d}}\right)\phi_{i,j,c}^{t-1} + (p_{i,j}-q_{i,j})\frac{\partial \phi_{i,j,c}^{t-1}}{\partial x_{i,d}}\Bigg\} \quad (34)$$

We can rewrite 34 as:

$$4\sum_{j\neq i}\Bigg\{\left(\frac{\partial p_{i,j}}{\partial x_{i,d}}\phi_{i,j,c}^{t-1} + p_{i,j}\frac{\partial \phi_{i,j,c}^{t-1}}{\partial x_{i,d}}\right) - \left(\frac{\partial q_{i,j}^{t-1}}{\partial x_{i,d}}\phi_{i,j,c}^{t-1} + q_{i,j}\frac{\partial \phi_{i,j,c}^{t-1}}{\partial x_{i,d}}\right)\Bigg\} \quad (35)$$

We can break the computation up into 2 parts. For the positive half, we notice that:

$$\sum_{j\neq i}\left(\frac{\partial p_{i,j}}{\partial x_{i,d}}\phi_{i,j,c}^{t-1} + p_{i,j}\frac{\partial \phi_{i,j,c}^{t-1}}{\partial x_{i,d}}\right) = \sum_{j:p_{i,j}\neq 0}\left(\frac{\partial p_{i,j}}{\partial x_{i,d}}\phi_{i,j,c}^{t-1} + p_{i,j}\frac{\partial \phi_{i,j,c}^{t-1}}{\partial x_{i,d}}\right) \quad (36)$$

This is because $\frac{\partial p_{i,j}}{\partial x_{i,d}} = 0 \iff p_{i,j} = 0$. As is done in (van der Maaten, 2013), we can use a sparse P matrix to reduce this computation to $O(n\log n)$.

For the negative half, we assume that we computed a quad-tree as per (van der Maaten, 2013). We can approximate this term using the summary embedding per quad-tree cell (denoted as y_{cell}):

$$\sum_{j\neq i}\left(\frac{\partial q_{i,j}^{t-1}}{\partial x_{i,d}}\phi_{i,j,c}^{t-1} + q_{i,j}\frac{\partial \phi_{i,j,c}^{t-1}}{\partial x_{i,d}}\right) \approx \sum_{cell\in cells} N_{cell}\left(\frac{\partial q_{i,cell}^{t-1}}{\partial x_{i,d}}\phi_{i,cell,c}^{t-1} + q_{i,cell}\frac{\partial \phi_{i,cell,c}^{t-1}}{\partial x_{i,d}}\right) \quad (37)$$

Where:

$$N_{cell} = \text{Number of points in the cell}$$

$$y_{cell} = \frac{1}{N_{cell}}\sum_{y_k\in cell} y_k$$

$$\phi_{i,cell,c}^{t-1} = (y_{i,c}^{t-1} - y_{cell,c}^{t-1})(1+||y_i^{t-1} - y_{cell}^{t-1}||^2)^{-1}$$

$$Z = \sum_i\sum_{cell\in cells} N_{cell}(1+||y_i^{t-1} - y_{cell}^{t-1}||^2)^{-1}$$

$$q_{i,cell} = \frac{(1+||y_i^{t-1} - y_{cell}^{t-1}||^2)^{-1}}{Z}$$

$$\frac{\partial \phi_{i,cell,c}^{t-1}}{\partial y_{i,c'}} = (1+||y_i^{t-1} - y_{cell}^{t-1}||^2)^{-1}1_{c=c'} - 2\phi_{i,cell,c}\cdot\phi_{i,cell,c'}$$

$$\frac{\partial q_{i,cell}^{t-1}}{\partial y_{i,c}} = -2q_{i,cell}\left(\phi_{i,cell,c} - 2\sum_{cell'\in cells} N_{cell'}q_{i,cell'}\phi_{i,cell',c}\right)$$

We can improve the efficiency of this calculation by reusing our quad-tree and Z term computed during the t-SNE objective calculation.

C. Synthetic Data Experiment

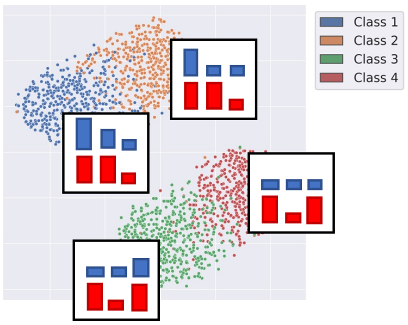

Fig. 4. The scatterplot of the t-SNE embeddings fit to synthetic data where "Effect 1" = 6 and "Effect 2" = 3, where each embedded datapoint is coloured by its class label. For each class and for the first 3 features, we display the feature value (top, in blue) and absolute value of the expected class-averaged attribution (bottom, in red). Note that we do not include the other 7 features since they are not enriched in any class, and we don't expect them to have high attribution.

We generated 10 dimensional random normal data containing 4 classes. Each class was distinguished by translation of a feature by a fixed amount (enrichment). We refer to this level of enrichment as "Effect 1". Feature 1 of class 1 and 2 were always enriched relative to class 3 and 4. Feature 2 was enriched by a fixed amount in class 1 relative to class 2, and feature 3 was enriched by the same amount in class 3 relative to class 4. See figure 4 for an illustration of this. We refer to this level of enrichment as "Effect 2". We varied "Effect 1" from 2,4,6 and "effect 2" from 1,2,3,5. We set "Effect 1" > "Effect 2" to make the structure heirarchical. We generated 10 datasets per combination and ran t-SNE with 10 different initializations on each of these. For each dataset, we averaged the attributions over each t-SNE and over all datapoints from the same class and took the absolute value.

Based on the data generating regime, we would expect the following features to have the highest attributions:

1. feature 1 for all classes
2. feature 2 for class 1 and 2
3. feature 3 for class 3 and 4

We performed a Mann-Whitney U-test to detect if the class-averaged attributions of these significant features were significantly greater then those of the remaining features. We performed our analysis on attributions coming from classes 1, 2, 3 and 4. From table S1 we can see that the class averaged attributions are significantly higher for the known important features versus the rest. We note that this holds even for class 4, which has no feature enrichment.

Putting this Experiment into Biological Context

The synthetic datasets were chosen to demonstrate that, if a small number of features are causing the observed structure, then our method will (mostly) identify those features. We do not guarantee that this adequately represents all real-world data or covers a wide range of scenarios. While the synthetic datasets contain cluster structure, we also envision synthetic datasets that contain trajectory structure (which is often found in single-cell transcriptomics data). t-SNE tends to have difficulty modeling such data, so remains to be seen how our method would perform in that context.

D. Perturbation Corruption Experiments

We repeated the local, class-based, and global level attribution experiments by permuting the values of each feature for all samples whose feature was to be corrupted. The results are presented in tables S5, S6 and S7. Note that the results are very similar to figures 2, except that the improvement from using feature-based removal is more pronounced on the individual level. We believe that this is an artifact of the corruption process, since permuting large values with each-other would have a more detrimental effect on the t-SNE versus permuting smaller values. Ideally, we would like to have "removed" the features, but we could only do so for the global level attribution experiments.

E. Using t-SNE Attributions For Quality Control

As mentioned before, our initial SARS-Cov-2 encoding scheme did not yield t-SNE embeddings that clustered based on the WHO designations. See in figure 5 A for a scatterplot of the t-SNE embeddings. That coding scheme included an additional column per position that was set to 1 if the position matched the reference and was set to 0 otherwise. Therefore, we can infer which positions are missing based on if all corresponding columns are 0. This motivated us to investigate the cause of this, providing another realistic use case for our methodology.

We first ran a DBSCAN clustering on those t-SNE embeddings. We then averaged the attributions of all the points within each cluster. We suspected that the cause of the malformed t-SNE embeddings was due to data missingness. We noticed that we could leverage our attributions to provide some evidence for this.

For each cluster, we investigated the relationship between the average attributions at each position and the frequency of missingness for each datapoint. If the missingness did not affect the cluster position, then we would expect a low correlation here. We found that this correlation was high for some clusters and low for others. We suspected that clusters containing the most missing positions would be the most impacted by the missingness (i.e. have the strongest positive correlation between attribution size and missingness frequency). Indeed, when we plot these quantities against each-other for all 20 clusters, we observed exactly this (see figure 5 B). Finally, since we had the missingness data available to us, we plotted the frequency of missingness per sequence and found that several clusters did appear to be highly enriched in missingness. In particular, clusters 1, 8, 11, 12, and 13 contain significantly high amounts of missingness, without a dominant lineage. The correlations of these clusters are 0.89, 0.81, 0.76, 0.76, and 0.85 respectively.

We note that removing the reference columns is equivalent to doing a kind of imputation where we substitute the missing data values for the reference values. When we recomputed the t-SNE after doing imputation we found that the embeddings clustered based on lineage, providing further evidence towards our initial suspicion. This can be seen in figure 3.

While we could have done this analysis using just the missingness information available to us, we emphasize that the attributions still would have correctly identified the positions that are often missing, providing a signal that would be useful here.

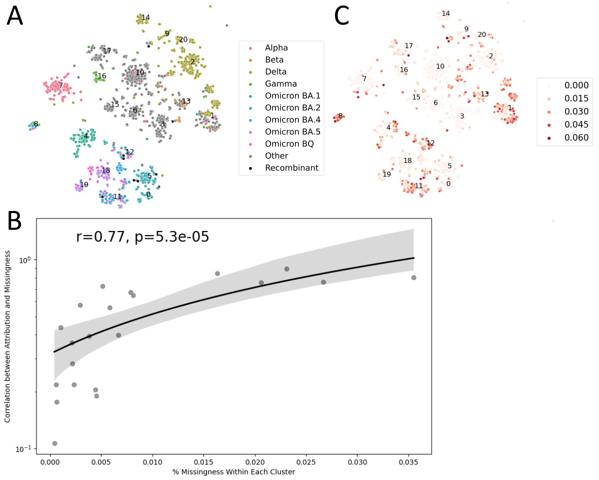

Fig. 5. Using Attributions of t-SNE to identify the cause of malformed embeddings. A: the malformed SARS-CoV-2 t-SNE embeddings. The centroid of each DBSCAN cluster is labelled with the cluster number. B. The correlation between the cluster-averaged attribution (y-axis) and the frequency of missing positions per cluster (x-axis). We also plot a line of best fit and display the correlation and p-value. Note that the y-axis is log-scaled. C: The proportion of missingness of each sequence displayed over the t-SNE embedding scatterplot.

F. Twenty News Groups Application

To demonstrate that our method works for data coming from very different modalities, we performed an analysis on the twenty newsgroups dataset. This dataset consists of ≈18000 newsgroups posts on 20 topics. For our purposes we further coarse grained out topic categories into the following: "Vehicles", "Sports", "Computers", "Ads", "Religion", "Politics", "Medicine" and "Space". For each post, we removed all punctuation, "stop words", and any words containing non-alphabet characters. We used the nltk python package for our data pre-processing (Bird et al., 2009).

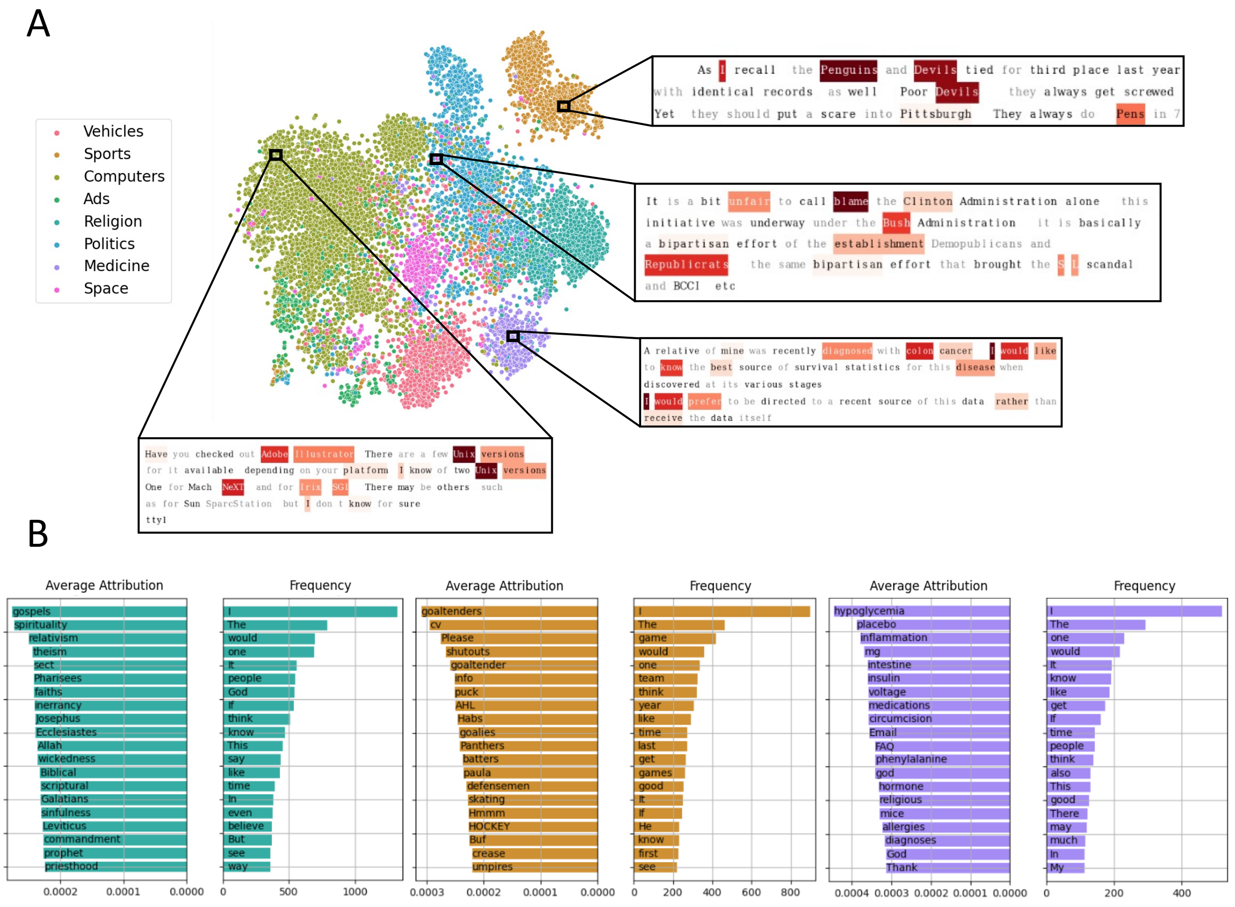

Fig. 6. t-SNE attribution analysis performed on the 20 NewsGroups dataset. A. The t-SNE group according to the conversation topic, as expected. When looking at attributions per conversation, we see that the highest attribution words tend to be strongly associated with the conversation topics. B. The average attributions per words (left side of bar plot) compared with the highest frequency words (right side), labelled with the word. The bidirectional bar plots use the same topic color mapping as the t-SNE.

We computed the document vector by taking the average of Word2Vec document embeddings (Mikolov et al., 2013) gensim implementation (Rehurek and Sojka, 2011). We performed our analysis on a random subset of 12000 posts, keeping only sentences with at least 25 words and at most 750. We projected our 300 dimensional document embeddings into 50 dimensions using PCA.

We performed t-SNE using the same hyperparameters as was done with the other datasets. Figure 6 A shows that, for the most part, posts from the same topic clustered together. We also see that the attributions highlighted words that were high associated with the post topic. Similarly, when we averaged the attributions per topic, we found that the highest attributed words consisted of jargon specific to the topic, or other words that were similarly highly associated with the topic. In figure 6 B we contrast the highest scoring words with the highest frequency words. Not surprisingly, the highest frequency words consisted of popular, generic words that were largely non-topic-specific.

G. Gradient Computation Details

We performed our t-SNE experiments using the following hyper-parameters:

1. perplexity = 30
2. number of iterations = 1000
3. the number of iterations of early exaggeration = 250
4. early exaggeration = 4
5. learning rate = 500

We did not experiment with other t-SNE hyper-parameter settings. We processed the attributions in the following way: first we extracted the attributions computed at the 250th step. We then removed any NaNs and clipped these so they would be in the range of -1 and 1. Finally, to convert the attributions with respect to the PC variables to those of the original inputs, we multiplied them by the PCA loadings matrix. When reporting attribution values or averages, we report the absolute value of the attribution(s).

Numerical Instabilities

We note that our proposed attribution computations are generally numerically stable. However, a small number of

gradients may be very large in magnitude (and possibly represented as NaNs). For example, for one of our MNIST runs, 0.36% of attributions were > 1 (0.19% > 10) and 0.36% were NaN. For our SARS-CoV-2 data, 0.042% of attributions were NaNs and 1.07% of the data was > 1 in absolute value (with 0.09% were greater than 10).

Attributions At Each t-SNE Iteration

For each experiment, we used the gradient of the t-SNE computed at step 250 for our attribution value (at the end of the early exaggeration phase). When we compared class-averaged attributions for each dataset, we found that attributions computed using gradients at this step best captured the globally-relevant features most often. This was especially apparent in our simulated data experiments, where the ground truth features were known. We hypothesize that this occurs due to the increased weight on the attractive forces during the early exaggeration phase. In the subsequent optimization steps, we found that the class-averaged gradients became uniform. We hypothesize that this is due to the increased weight on the repulsive forces. When this occurs the optimization adds more priority towards placing embeddings away from nearby neighboring ones. Inspecting the attributions computed during these later optimization steps may yield useful insights, but we leave such analysis for future work. See figure 7 for a visualization of the class-averaged attributions computed over each step of the t-SNE optimization.

Benchmarking Experiments

To assess the computational speed of our algorithm, we performed a series of benchmarking experiments on our t-SNE gradients implementation as well as our implementation of ordinary t-SNE. We performed this benchmarking experiment on synthetic datasets containing the same structure as described in figure 4. For all experiments, we only ran t-SNE for the first 250 steps, using the Barnes-Hut approximation. Both experiments were performed on a compute node provided by the Digital Research Alliance of Canada containing 16 CPU cores with 24GB RAM.

In our first experiment, we varied the number of features from 20 to 380 in increments of 20, keeping the number of samples fixed at 1000. In our second experiment, we varied the number of samples from 1000 to 20000 in increments of 1000, keeping the number of features fixed at 10. Not surprisingly, we found that both implementations scaled roughly loglinearly with respect to number of samples. As expected, we found that our gradient computations scaled roughly linearly with respect to number of input features. Note that the usual t-SNE is unaffected by the number of input features (except for the computation of P at the beginning), and so we did not benchmark this relationship. See figure 8 for more details.

Qualitatively, we find that the attribution computation is considerably slower versus usual t-SNE. For example, our t-SNE implementation took around 2 seconds to compute, but the attributions took anywhere from 5 seconds to 2 minutes to compute. Despite this, our algorithm can in theory be applied to datasets of arbitrary size. This is since t-SNE is usually fitted on PC-transformed data with a small dimension of fixed size (usually 50-100). Therefore, the linear complexity with respect to the number of features should not be very noticeable in practice.

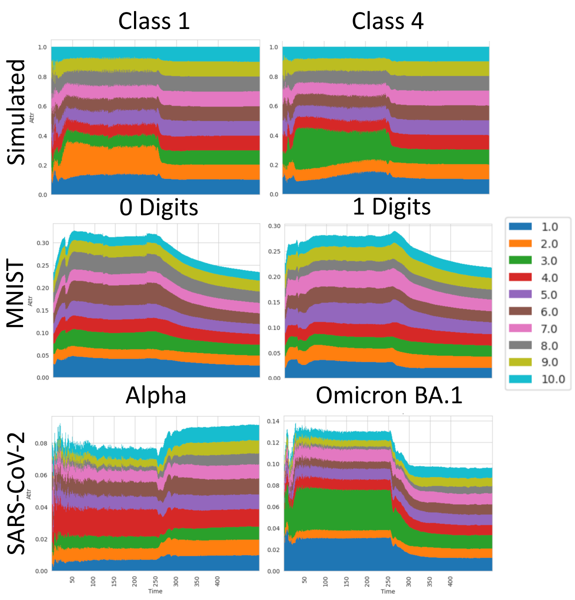

Fig. 7. t-SNE Attributions at each step for each dataset. For each dataset, we select 2 classes displayed the (absolute value of the) class-averaged attributions across the first 500 steps of the t-SNE algorithm. We display the first 10 features (for datasets with more then 10 features, we ignore the rest here). (Top) The simulated dataset. We display Class 1 and 4. Note that for Class 1, attributions of feature 1 and 2 are significantly larger then the rest, as expected. Likewise for Class 4, the features with the largest attributions are the ground truth features. (Middle) For the 0 and 1 digit classes of MNIST, we see that certain features grow relative to the rest until step 250, after which the feature attribution sizes become increasingly uniform. (Bottom) We observe the same pattern for the SARS-CoV-2 data.

Limitations and Unexpected Results of Gradient Attributions

We briefly discuss some limitations of our our t-SNE attribution method that we observed during our simulated data experiments. In the following two cases, the attributions did not identify the relevent features consistently (or at all):

1. When the dataset was small ($<$ 1000 points).
2. When the feature enrichment was very large. For example the attributions of the simulated data with “Effect 1” $= 8$ performed poorly.

Furthermore, we note that, unexpectedly, the size of attributions for feature 1 was generally smaller then for feature 2 or 3, despite that “Effect 1” was always larger then “Effect 2”.

The cause of any of these limitations/unexpected results are not explored at depth here. We leave as future work a more rigorous characterization of these limitations and how to improve our method to resolve them.

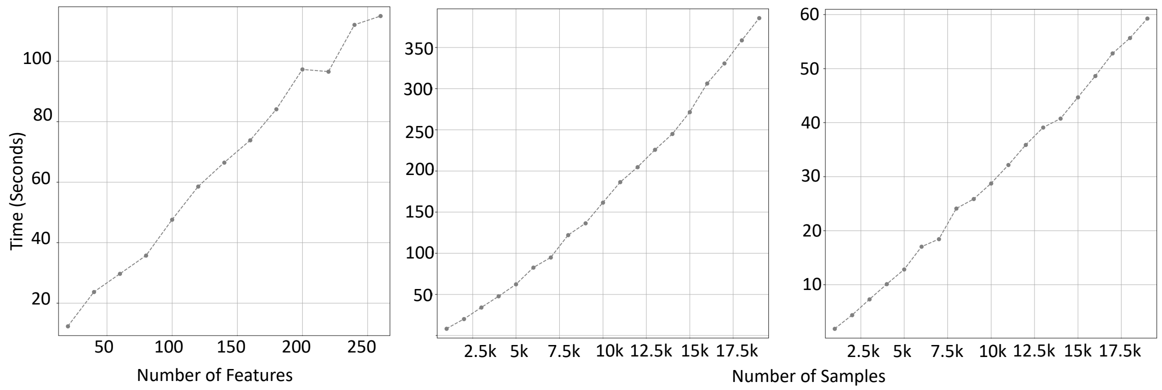

Fig. 8. Benchmark experiments. (*Left*) complexity of our t-SNE attribution implementation with respect to the number of features. (*Center*) complexity with respect to the number of input samples. (*Right*) complexity with respect to number of input samples for t-SNE.

References

Bird, S., Klein, E., and Loper, E. (2009). *Natural language processing with Python: analyzing text with the natural language toolkit.* " O'Reilly Media, Inc.".

Mikolov, T., Sutskever, I., Chen, K., Corrado, G., and Dean, J. (2013). Distributed representations of words and phrases and their compositionality. In *Proceedings of the 26th International Conference on Neural Information Processing Systems - Volume 2*, NIPS'13, page 3111–3119, Red Hook, NY, USA. Curran Associates Inc.

Rehurek, R. and Sojka, P. (2011). Gensim–python framework for vector space modelling. *NLP Centre, Faculty of Informatics, Masaryk University, Brno, Czech Republic*, 3(2).

Supplemental Tables

We present the tables for the Synthetic data and MNIST attribution validation experiments here.

Table S1. Results of synthetic data experiment

Effect 1	Effect 2	class	Mean of Sig. Attrs	Mean of Non-Sig. Attrs	Attr. P-Value
2	1	1	0.0044 ± 0.0027	0.0007 ± 0.0006	< 1e-4
2	1	2	0.0041 ± 0.0022	0.0007 ± 0.0005	< 1e-4
2	1	3	0.0054 ± 0.0039	0.0009 ± 0.0007	< 1e-4
2	1	4	0.0046 ± 0.0030	0.0007 ± 0.0006	< 1e-4
4	1	1	0.0083 ± 0.0067	0.0018 ± 0.0012	< 1e-4
4	1	2	0.0101 ± 0.0071	0.0016 ± 0.0009	< 1e-4
4	1	3	0.0105 ± 0.0090	0.0018 ± 0.0014	0.0002
4	1	4	0.0095 ± 0.0065	0.0016 ± 0.0010	< 1e-4
4	2	1	0.0169 ± 0.0065	0.0025 ± 0.0016	< 1e-4
4	2	2	0.0125 ± 0.0054	0.0017 ± 0.0013	< 1e-4
4	2	3	0.0174 ± 0.0058	0.0020 ± 0.0014	< 1e-4
4	2	4	0.0130 ± 0.0038	0.0016 ± 0.0011	< 1e-4
4	3	1	0.0335 ± 0.0265	0.0033 ± 0.0026	< 1e-4
4	3	2	0.0248 ± 0.0123	0.0022 ± 0.0019	< 1e-4
4	3	3	0.0333 ± 0.0254	0.0037 ± 0.0027	< 1e-4
4	3	4	0.0248 ± 0.0117	0.0024 ± 0.0017	< 1e-4
6	1	1	0.0059 ± 0.0040	0.0023 ± 0.0014	< 1e-4
6	1	2	0.0059 ± 0.0045	0.0027 ± 0.0017	0.0020
6	1	3	0.0079 ± 0.0047	0.0032 ± 0.0022	< 1e-4
6	1	4	0.0054 ± 0.0029	0.0025 ± 0.0018	< 1e-4
6	2	1	0.0186 ± 0.0183	0.0034 ± 0.0023	0.0010
6	2	2	0.0161 ± 0.0132	0.0028 ± 0.0019	< 1e-4
6	2	3	0.0211 ± 0.0179	0.0032 ± 0.0024	< 1e-4
6	2	4	0.0154 ± 0.0118	0.0027 ± 0.0017	< 1e-4
6	3	1	0.0241 ± 0.0231	0.0030 ± 0.0024	< 1e-4
6	3	2	0.0153 ± 0.0171	0.0020 ± 0.0013	< 1e-4
6	3	3	0.0244 ± 0.0227	0.0032 ± 0.0022	< 1e-4
6	3	4	0.0143 ± 0.0120	0.0021 ± 0.0016	< 1e-4
6	5	1	0.0087 ± 0.0075	0.0025 ± 0.0014	0.0005
6	5	2	0.0077 ± 0.0044	0.0023 ± 0.0011	< 1e-4
6	5	3	0.0083 ± 0.0071	0.0022 ± 0.0012	0.0002
6	5	4	0.0076 ± 0.0049	0.0022 ± 0.0011	< 1e-4

Table S2. MNIST Experiment individual-level using mean averaging feature corruption

Index	Correlation	Adjusted RAND Index	KNN Preservation
Random	0.70 ± 0.0715	0.75 ± 0.0290	0.42 ± 0.0017
Attribution > 0	0.58 ± 0.0772	0.62 ± 0.0154	0.33 ± 0.0032
Attribution	0.55 ± 0.0587	0.59 ± 0.0261	0.28 ± 0.0035
Feature Value	0.32 ± 0.0510	0.38 ± 0.0129	0.30 ± 0.0006
Attribution · Feature Value	0.35 ± 0.0361	0.36 ± 0.0240	0.20 ± 0.0024

Table S3. MNIST Experiment class-level using mean averaging feature corruption

Index	Correlation	Adjusted RAND Index	KNN Preservation
Random	0.76 ± 0.0625	0.76 ± 0.0507	0.62 ± 0.0029
Top Laplace Score Per Class (using P)	0.56 ± 0.0779	0.72 ± 0.0476	0.51 ± 0.0017
Top Laplace Score Per Class (using Q)	0.62 ± 0.0643	0.72 ± 0.0444	0.51 ± 0.0028
Top Attribution Per Class	0.49 ± 0.0992	0.69 ± 0.0434	0.49 ± 0.0034
Top Feature Value Per Class	0.50 ± 0.0657	0.69 ± 0.0454	0.50 ± 0.0019
Top Attribution · Feature Value Per Class	0.52 ± 0.0589	0.69 ± 0.0363	0.50 ± 0.0027

Table S4. MNIST Experiment global-level using feature removal corruption

Index	Correlation	Adjusted RAND Index	KNN Preservation
Random	0.86 ± 0.0326	0.81 ± 0.0458	0.64 ± 0.0037
Top Fisher Score	0.68 ± 0.0588	0.72 ± 0.0284	0.54 ± 0.0026
Top PC	0.52 ± 0.0521	0.66 ± 0.0425	0.50 ± 0.0020
Top Laplace Score (Using P)	0.53 ± 0.0509	0.68 ± 0.0408	0.51 ± 0.0013
Top Laplace Score (Using Q)	0.53 ± 0.0542	0.68 ± 0.0387	0.51 ± 0.0013
Top Feature Value	0.52 ± 0.0521	0.66 ± 0.0425	0.50 ± 0.0020
Attribution	0.55 ± 0.0665	0.67 ± 0.0434	0.50 ± 0.0042
Attribution · Feature Value	0.51 ± 0.0581	0.67 ± 0.0427	0.50 ± 0.0026

Table S5. MNIST Experiment individual-level using permutation feature corruption

Index	Correlation	Adjusted RAND Index	KNN Preservation
Random	0.59 ± 0.0760	0.64 ± 0.0189	0.33 ± 0.0011
Attribution > 0	0.46 ± 0.0535	0.51 ± 0.0203	0.23 ± 0.0044
Attribution	0.42 ± 0.0457	0.45 ± 0.0320	0.18 ± 0.0043
Feature Value	0.07 ± 0.0140	0.08 ± 0.0026	0.08 ± 0.0004
Attribution · Feature Value	0.13 ± 0.0249	0.14 ± 0.0062	0.08 ± 0.0006

Table S6. MNIST Experiment class-level using permutation feature corruption

Index	Correlation	Adjusted RAND Index	KNN Preservation
Random	0.75 ± 0.0310	0.77 ± 0.0508	0.47 ± 0.0018
Top Laplace Score Per Class (using P)	0.38 ± 0.0263	0.44 ± 0.0350	0.17 ± 0.0009
Top Laplace Score Per Class (using Q)	0.44 ± 0.0313	0.52 ± 0.0413	0.19 ± 0.0022
Top Attribution Per Class	0.28 ± 0.0528	0.42 ± 0.0346	0.18 ± 0.0070
Top Feature Value Per Class	0.18 ± 0.0115	0.26 ± 0.0195	0.13 ± 0.0010
Top Attribution · Feature Value Per Class	0.17 ± 0.0301	0.26 ± 0.0191	0.14 ± 0.0038

Table S7. MNIST Experiment global-level using permutation feature corruption

Index	Correlation	Adjusted RAND Index	KNN Preservation
Random	0.78 ± 0.0357	0.78 ± 0.0483	0.46 ± 0.0022
Top Fisher Score	0.57 ± 0.0411	0.62 ± 0.0364	0.24 ± 0.0016
Top PC	0.19 ± 0.0321	0.30 ± 0.0207	0.14 ± 0.0014
Top Laplace Score (Using P)	0.19 ± 0.0302	0.31 ± 0.0240	0.15 ± 0.0009
Top Laplace Score (Using Q)	0.19 ± 0.0302	0.31 ± 0.0240	0.15 ± 0.0011
Top Feature Value	0.19 ± 0.0321	0.30 ± 0.0207	0.14 ± 0.0014
Attribution	0.27 ± 0.0485	0.35 ± 0.0401	0.16 ± 0.0076
Attribution · Feature Value	0.20 ± 0.0293	0.30 ± 0.0232	0.15 ± 0.0014
